# Native Renal Arteries Denervation as a Therapy of Refractory Hypertension in Patient after Heart and Kidney Transplantation—5 Years of Observation

**DOI:** 10.3390/jcm12175458

**Published:** 2023-08-23

**Authors:** Justyna Pilch, Jakub Mizera, Krzysztof Wiśnicki, Marcin Protasiewicz, Jacek Kurcz, Sławomir Zmonarski, Patryk Wawrzonkowski, Krzysztof Letachowicz, Dorota Kamińska, Tomasz Gołębiowski, Michał Zakliczyński, Magdalena Krajewska, Mirosław Banasik

**Affiliations:** 1 Faculty of Medicine, Wroclaw Medical University, 50-368 Wroclaw, Poland; jakub.mizera@student.umw.edu.pl; 2 Clinical Department of Nephrology and Transplantation Medicine, Wroclaw Medical University, 50-556 Wroclaw, Poland; krzysztof.e.wisnicki@gmail.com (K.W.); slawomir.zmonarski@umw.edu.pl (S.Z.); p.wawrzon@gmail.com (P.W.); krzysztof.letachowicz@umw.edu.pl (K.L.); dorota.kaminska@umw.edu.pl (D.K.); tomasz.golebiowski@umw.edu.pl (T.G.); magdalena.krajewska@umw.edu.pl (M.K.); m.banasik@interia.pl (M.B.); 3 Clinical Department of Cardiology, Wroclaw Medical University, 50-556 Wroclaw, Poland; marcin.protasiewicz@umw.edu.pl; 4 Institute of Heart Diseases, University Hospital, 50-556 Wroclaw, Poland; michal.zakliczynski@umw.edu.pl; 5 Clinical Department of Radiology, Wroclaw Medical University, 50-556 Wroclaw, Poland; jacek.kurcz@umw.edu.pl; 6 Clinic of Cardiac Transplantation and Mechanical Circulatory Support, Wroclaw Medical University, 50-556 Wroclaw, Poland

**Keywords:** renal arteries denervation, refractory hypertension, innovative treatment, kidney transplantation, heart transplantation

## Abstract

This case report describes a 59-year-old male patient after heart and kidney transplantation, subsequently diagnosed with refractory hypertension since implemented antihypertensive pharmacotherapy consisting of six agents did not provide a substantial therapeutic response. Elevated blood pressure and its impact on a hypertrophied transplanted heart and impaired renal graft function have led to a significant deterioration in the patient’s cardiovascular risk profile. To address this issue, a native renal arteries denervation was performed. It resulted in a noteworthy decrease in both systolic and diastolic pressure values, thus manifesting a positive hypotensive effect. Furthermore, a sustainable reduction of left ventricular mass and stabilization in kidney graft function were noticed. The presented case provides evidence that renal denervation can be an efficacious complementary treatment method in individuals who received kidney and heart grafts as it leads to a decrease in cardiovascular risk.

## 1. Introduction

Hypertension remains a pressing health issue in transplant recipients’ populations worldwide. Despite the advancements in surgical techniques and pharmacological treatment methods, kidney graft recipients are still particularly prone to cardiovascular morbidity and mortality due to the development of hypertension. Consistently elevated blood pressure not only is the main cause of death in kidney transplant recipients but also poses a great threat of non-immunological graft failure. Based on the available literature, approximately 70% to 90% of patients after kidney transplantation suffer from hypertension [1], among whom 34.2% require the concomitant implementation of three antihypertensive agents, while 30.6% necessitate the simultaneous use of four or more antihypertensive drugs. Notably, treatment resistance is observed in approximately 20% of the recipient population [2], out of which a noteworthy number of patients are diagnosed with refractory hypertension characterized by the blood pressure remaining uncontrolled despite the use of ≥5 antihypertensive agents of different classes at their recommended doses [3].

Since hypertension is a multifactorial condition with several underlying etiologies, therapy can be aimed at different pathomechanisms. One of the potential therapeutic targets is excessive renal sympathetic activity being a response to upheavals in the organism [4]. Kidneys have been widely recognized for their pivotal role in the control of arterial blood pressure, primarily by modulating the total body fluid volume, maintaining sodium homeostasis, and stimulating the synthesis and secretion of vasoconstrictive hormones. All of the aforementioned processes influence global arterial smooth muscle tone, which is the primary hemodynamic determinant of blood pressure. However, available data indicate that renal sympathetic overactivity is a key player in the onset and progression of hypertension. The renal sympathetic nerves provide innervation to the three major neuroeffectors within the kidney. Increased renal sympathetic activity results in excessive renin secretion by juxtaglomerular granular cells in the course of β-1 adrenoceptors stimulation, decreased urinary sodium excretion in renal tubules, higher renal sodium reabsorption by the stimulation of alpha-1 b receptors and diminished renal blood flow as a result of alpha-1 a receptors stimulation. A comprehensive understanding of the renal system and its physiological role in maintaining homeostasis within the human organism is undeniably crucial in the process of formulating and enhancing therapeutic interventions for hypertension. Therefore native renal denervation has emerged as a potentially promising therapeutic strategy as it disrupts the nerves in the renal artery, thus inhibiting excessive sympathetic activity [5].

## 2. Materials and Methods

A systematic review of the literature published until June 2023 was performed in PubMed and Google Scholar databases for renal denervation case studies and meta-analyses. To identify contingent valuation studies related to renal denervation procedures, a search was carried out using a combination of medical terms: renal sympathetic denervation, renal denervation case studies, hypertension renal denervation, renal denervation case reports.

The initial search yielded 3896 articles. Duplicates were ruled out, and then each article was reviewed for the following inclusion criteria: English language, case reports, and pertinence to renal denervation procedures. In consequence, 22 papers especially relating to our study were selected to be mentioned in the manuscript.

We confirm that informed consent regarding the procedure and the data publication was obtained from the patient.

## 3. Case Report

We report on a case study of a 59-year-old male patient suffering from refractory hypertension who experienced a myocardial infarction in 1990, underwent coronary artery bypass grafting (CABG) in 1998, and received a heart transplant in 2010 due to progressive heart failure. Additionally, the patient received a kidney transplant in 2011. The immunosuppressive regimen following these procedures consisted of steroids, calcineurin inhibitor (CNi), and mycophenolate mofetil (MMF). Due to the long-lasting treatment, the patient developed type 2 diabetes mellitus and required insulin therapy in the early stages, which afterward was transitioned to oral therapy. The effectiveness of the kidney transplant, although suboptimal, remained stable with a creatinine level of approximately 1.8 mg/dL and an estimated glomerular filtration rate (eGFR) of 41 mL/kg/min. Despite the graft function being sufficient, the patient developed uncontrollable high blood pressure with values reaching up to 180/90 mmHg. The implemented antihypertensive treatment consisted of bisoprolol, clonidine, nitrendipine, ramipril, doxazosin, and furosemide, administered at the doses recorded in Table 1. However, the pharmacotherapy did not provide a sufficient therapeutic outcome.

The pre-denervation assessment of the transplanted heart was performed with magnetic resonance imaging (MRI) and revealed a glaring left ventricular hypertrophy (LVH) with its mass calculated at 256.4 g. The ejection fraction equaled 74.4% which corresponded with the hypertrophy. Since uncontrolled hypertension is a primary cause of LVH and the threat of progression of kidney graft malfunction, a need for a different and more effective treatment method emerged. Given the prior described successful case of a renal transplant patient in whom denervation had resulted in a decrease in blood pressure levels, along with the depletion of non-invasive treatment methods, the decision to perform the intervention was established. Initially, the assessment of the patient’s native renal arteries was carried out with MRI to prevent any potential harm from contrast used in computed tomography (CT). The denervation was performed in September 2014 and undertaken through the right femoral artery, with intravenous application of heparin (ACT > 250 s). An ablative catheter was introduced into the renal artery using a 6 F guiding catheter and the deployment of eight sets of energy was performed bilaterally. To the best of our knowledge, this was the first native renal arteries denervation procedure in a patient after kidney and heart transplantation.

Treatment verification during 1-year postoperative observation revealed an improvement in blood pressure control and a notable decrease of 14 and 11 mmHg in systolic and diastolic pressure values, respectively (168/88 mmHg vs. 154/77 mmHg). The results were obtained by 24-h ambulatory blood pressure monitoring (ABPM). Moreover, MRI evaluation demonstrated a significant reduction in the left ventricular wall thickness in the septal and free wall segments, followed by sustainable left ventricular mass reduction from 256.4 g to 215.6 g (Table 2).

During the 5-year observation, mean arterial pressure was calculated at 163/77 mmHg. MRI presented no alterations in left ventricular mass and a slight decrease in the average left ventricular wall thickness calculated across all segments, in comparison to the previous measurements.

A significant reduction in left ventricular stroke volume was noted, however, the EF was calculated at 65.2%, which means it finally reached the desirable range of 50–70%. In the case of our patient, this reduction in EF corresponded with a reduction in LVH and normalization in heart function. Renal graft function parameters remained stable, with minor alterations of creatinine levels reaching up to 2.0 mg/dL on average (Table 2).

## 4. Results

Both 1-year and 5-year follow-up protocols proved denervation to be beneficial for the patient. In the presented case, the intervention has led to reductions in both systolic and diastolic pressures—a decrease of 14 mmHg in systolic pressure and 11 mmHg in diastolic pressure after 1 year, followed by reductions of 5 mmHg in systolic pressure and 11 mmHg in diastolic pressure after 5 years. These findings underscore a favorable hypotensive effect of the intervention (Table 2). However, the procedure did not allow us to significantly reduce drug antihypertensive polytherapy nor to achieve optimal blood pressure levels (Table 1). The observed reduction of left ventricular mass is believed to have a substantial impact on decreasing cardiovascular risk since it is commonly known as an adverse prognostic factor in patients suffering from hypertension. Furthermore, the intervention enabled the patient to achieve an optimal ejection fraction during a 5-year follow-up protocol, which aligns with the improved function of a transplanted heart. Regarding kidney transplant survival, we did not notice a significant decrease in graft function parameters, which implies the safety of the renal denervation procedure.

## 5. Discussion

Drug-resistant hypertension constitutes a tremendous issue in patients after kidney transplantation. Native renal arteries denervation as a complementary treatment to pharmacotherapy seems to be a promising solution.

Currently, at least three different percutaneous approaches to renal arteries denervation are actively investigated. Regardless of the chosen method, the first step consists of the insertion of a catheter into the renal artery through a small incision in the groin or wrist. Subsequently, the catheter is carefully guided to the targeted area. Once in position, diverse methods can be implemented to disrupt the nerves, including ultrasound, radiofrequency energy, or neurotoxin injections. Ultrasound techniques deliver usually 4 ultrasound-emitting sources mounted on a catheter together with an inflatable balloon system, which enables the irrigation of the catheter segment in contact with the arterial wall using a temperature-lowering solution. It sustains a cooler temperature within the lumen of the renal artery in comparison to the perivascular space. Radiofrequency ablation (RFA) is a commonly used technique and entails the use of a catheter equipped with typically 4 electrodes that generate heat through medium-frequency alternating current. The generated heat has a toxic effect on the nerves surrounding the renal artery within the heat energy field which extends up to 7 mm from the lumen of the renal artery. Concomitantly, the heat seems to be well tolerated by the arterial wall. The injections of neurolytic agents are performed with the use of a small catheter containing 3 microneedles embedded within it concentrically. Once the catheter is properly positioned, the microneedles are extended and penetrate through the renal artery wall into the perivascular space. Subsequently, a small volume of the neurotoxin is simultaneously injected through all the needles—around 0.6 mL of absolute ethanol in total. It selectively destroys nerve fibers and disrupts their transmission [6].

The efficacy and effectiveness of renal denervation have been the subject of scrutiny among numerous researchers. Initially, upon its introduction, there was widespread optimism regarding the procedure, leading to its implementation in a diverse range of patients with varying underlying conditions [7]. However, the results of the randomized controlled SYMPLICITY HTN-3 trial published in 2014 raised doubts about the suitability of the procedure for the whole patient population, thus generating controversy. Bhatt et al. screened a population of 535 patients with severe hypertension among whom 364 underwent renal denervation and 171 underwent the sham procedure. In both groups, patients were examined before the denervation and the mean systolic blood pressure was calculated at 188 mmHg. Moreover, patients took an average of 5 antihypertensive medications. A 6-month follow-up examination revealed a decrease in systolic pressure in both groups, however, the differences were not statistically significant (14.13 mmHg after denervation vs. 11.74 mmHg after the sham procedure). Interestingly, the procedure was found to be successful in three particular groups of patients: individuals of other races than black, below the age of 65, and with eGFR reaching at least 60 mL/min/1.73 m^2^ [8]. While the 6-month follow-up of the randomised SYMPLICITY HTN-3 trial showed safety but not necessarily efficacy of the catheter-based renal artery denervation procedure, the final report published after 36 months of observation provides new interesting findings. The data obtained in the course of the final follow-up examinations support the evidence regarding the safety of the procedure. Moreover, patients on whom the renal denervation was originally randomly performed presented larger reductions in blood pressure values and better blood pressure control, in comparison to patients who were randomly assigned to a sham control [9].

Nowadays, renal denervation is once again in the spotlight, with several studies demonstrating its effectiveness, although limited to specific patient groups [7].

The first renal denervation performed in Korea as a therapeutic approach in the course of heart failure was described by Yang et al. The 44-year-old male patient exhibited a seven-year history of hypertension and presented uncontrolled blood pressure levels despite treatment with four different antihypertensive medications. To address this issue, a percutaneous renal denervation procedure was performed under local anesthesia with the use of an ablative catheter. Subsequently, a 6-month follow-up revealed a significant reduction in blood pressure, measured at 49 mmHg and 37 mmHg for systolic and diastolic blood pressure, respectively, accompanied by a decrease of 20/18 mmHg in 24-h ambulatory blood pressure monitoring. No noteworthy stenosis in either renal artery was revealed in the renal Doppler examination [10].

Pushpakumar et al. verified the effectiveness of renal denervation in individuals suffering from heart failure. The procedure was performed on animal models, hence it requires investigation in human studies. Researchers indicated that renal denervation can be successful in hypertension treatment since it reduces cardiac hypertrophy and has cardioprotective features. The observed cardioprotective effects may be attributed to the preservation of endothelial nitric oxide synthase (eNOS) activity, leading to improvements in vascular endothelial function. Moreover, the procedure demonstrated favorable outcomes for renal health by protecting against renal stress and apoptosis. As a result, the researchers suggest that renal denervation may be particularly beneficial for individuals with reduced left ventricular function who are at increased risk of cardiovascular complications [11].

The study conducted by Huang et al. on rabbit models with heart failure resulted in the conclusion that renal denervation prevents not only structural and functional remodeling of the heart but also arrhythmogenesis since it decreases QT interval and QT interval dispersion [12].

Yilmaz et al. have recently revealed significant findings indicating the efficacy of renal denervation in the patient suffering from multidrug-resistant hypertension, who had previously undergone renal artery stent implantation. Despite receiving a pharmacological treatment regimen comprising amlodipine (10 mg), doxazosin (8 mg), valsartan (320 mg), hydrochlorothiazide (25 mg), spironolactone (50 mg), and furosemide (40 mg), the antihypertensive effect was insufficient. In this particular case, catheter-based renal denervation was proved to be effective, leading to a significant reduction in blood pressure levels [13].

In the study conducted by Kario et al., the authors suggest renal denervation to be a potentially effective intervention for patients suffering from elevated morning and nighttime values of blood pressure. The study involved a group of 80 patients with an increased risk of cardiovascular events, such as myocardial infarction or stroke. In a 3-year follow-up ABPM examination, a noteworthy decrease in systolic pressure values was noted (reduction of 20.2 mmHg). Moreover, morning and nighttime systolic blood pressure was significantly lower (23.9 mmHg and 20.8 mmHg, respectively). Based on these results, the procedure has been proven to be beneficial for this particular group of patients since it led to a substantial reduction in cardiovascular risk profile [14].

The idea of renal denervation in post-kidney transplantation patients was introduced by Sas et al. in 2015, who were the first to perform this procedure in kidney graft recipient. Before the denervation, the patient suffered from uncontrolled refractory hypertension and despite the applied treatment consisting of amlodipine, clonidine, bisoprolol, doxazosin, and furosemide at their highest recommended doses, the antihypertensive effect was not satisfactory. A 3-year follow-up observation revealed a substantial decrease in blood pressure values (149/96 mmHg vs. 134/91 mmHg). Moreover, a significant decrease in left ventricular mass was noticed (577 g vs. 470 g). This case highlights the potential suitability of renal denervation as a therapeutic option for transplant recipients, as it not only exerts antihypertensive effects and protects against cardiac hypertrophy but also proves to be safe for the transplanted kidney without compromising its function [15].

During the last decade, several case reports indicated the potential role of renal denervation in combination with pulmonary vein isolation as a method for atrial fibrillation treatment. In a cohort of patients (ERADICATE—AF trial) presenting with paroxysmal atrial fibrillation and concomitant hypertension, the supplementation of renal denervation to catheter ablation in pulmonary veins demonstrated a statistically significant augmentation in the probability of achieving sustained freedom from atrial fibrillation after a 12-month follow-up, as compared to the employment of catheter ablation in pulmonary veins alone. Even though the findings are inquisitive, they require further investigation and confirmation [16].

Moreover, first-in-man robotic-assisted renal denervation was recently described by Bermpeis et al. The intervention was performed on a 43-year-old female suffering from drug-resistant hypertension. Ablation was performed in both right and left renal arteries with a total of 12 and 16 ablated spots, respectively. The entire procedure was carried out by the operator at the console and was well tolerated by the patient who was discharged the same day. As renal denervation is considered a complementary and not alternative therapy to pharmacological antihypertensive treatment, pharmacotherapy remained unmodified after the procedure. The outcome of the intervention was assessed during AMBP 2 months later and demonstrated a significant decrease of 20 mm Hg in mean systolic blood pressure [17] (Table 3).

### 5.1. Literature Limitations

This study, while providing valuable insights, is not without limitations.

While the literature review conducted for this study aimed to comprehensively survey the available research in the field, certain limitations should be acknowledged. Firstly, the search strategy primarily focused on peer-reviewed articles published in English, potentially leading to the omission of relevant studies published in other languages. This language bias might limit the inclusiveness of the review. Secondly, although efforts were made to include studies from a wide range of databases, it’s possible that some relevant studies were inadvertently excluded due to variations in indexing and database coverage.

### 5.2. Study Limitations

Despite the promising nature of our findings, it is important to acknowledge that the reduction in blood pressure (BP) values and the decrease of LVH may have arisen from factors other than the denervation procedure itself. Over the course of the follow-up period, alterations in pharmacotherapy were implemented (Table 1). While antihypertensive agent dosages increased during the initial year of observation, they were subsequently tapered at the 5-year follow-up. Additionally, a reduction in tacrolimus dosages was evident at the 5-year assessment, with a decrease from 12 mg per day to 6 mg per day. Given that calcineurin inhibitors (CNIs) have the potential to elicit hypertensive effects within the body, it remains possible that such alterations in medication regimens could have contributed to the observed decline in patients’ BP levels [18]. Moreover, LVH might also be a rare complication associated with tacrolimus administration, thus the decrease in its dosage could also result in reduction of left ventricular mass [19].

Finally, given that our patient underwent heart transplantation four years prior to renal denervation, it is possible that alterations in cardiac structure and function may have arisen as a consequence of cardiac re-innervation rather than the renal denervation itself. Cardiac re-innervation has been associated with the potential increase in haemodynamic perfusion within the coronary arteries and improved exercise tolerance. This restorative process can manifest itself even several years subsequent to the transplantation and may therefore have occurred in the case of our patient during the follow-up period [20].

## 6. Conclusions

To conclude, we consider renal arteries denervation to be a potentially promising complementary treatment procedure for refractory hypertension in patients after kidney and heart transplantation. Since, according to Lewington et al., the decrease in arterial pressure significantly reduces the risk of stroke, myocardial infarction, and cardiovascular mortality, it is essential to provide patients with the most effective treatment approach. Well-controlled hypertension not only reduces cardiovascular risk but also significantly improves long-term graft survival [21,22]. However, our endorsement of renal arteries denervation must be contextualized within the broader landscape of research. While we have cited supportive studies, it is essential to acknowledge that these primarily consist of case reports and small cohorts. Furthermore, we would like to highlight the inconclusive nature of large randomised trials, which have failed to consistently demonstrate the anticipated benefits. As such, the success and failure rates of renal denervation are both well-documented, underscoring the complexity of this intervention. In light of our own study, in which systolic blood pressure was reduced by only 5 mmHg at the 5-year follow-up compared to pre-intervention levels, we recognise the variability and unpredictability inherent in the results of renal arteries denervation. We also emphasise that while a trial of renal denervation may be reasonable in selected cases, it is crucial to acknowledge the equivocal outcomes and the fact that the safety of this intervention in specific and relatively vulnerable patient populations requires additional evidence. We anticipate these preliminary findings to serve as a stimulus for future investigations.

## Figures and Tables

**Table 1:** Pharmacotherapy administered before denervation and during 5 years of observation.

Medication Group	Medication	Before Denervation	1-Year Follow up	5-Year Follow up
Immunosuppresive	Prednisone	1 × 5 mg	1 × 5 mg	1 × 5 mg
	Tacrolimus	2 × 6 mg (12 mg)	2 × 5 mg (10 mg)	1 × 6 mg
	Mycophenolate mofetil	2 × 500 mg (1000 mg)	2 × 750 mg (1500 mg)	2 × 250 mg (500 mg)
Anti-hypertensive	Bisoprolol	2 × 2.5 mg (5 mg)	1 × 10 mg	1 × 5 mg
	Nitrendipine	2 × 20 mg (40 mg)	2 × 20 mg (40 mg)	2 × 20 mg (40 mg)
	Doxazosin	2 × 2 mg (4 mg)	1 × 2 mg	2 × 4 mg (8 mg)
	Clonidine	2 × 75 μg (150 μg)	3 × 75 μg (225 μg)	-
	Ramipril	1 × 2.5 mg	1 × 2.5 mg	-
	Furosemide	1 × 40 mg	1 × 40 mg	-
	Torasemide	-	-	1 × 10 mg
Metabolic	Glimepiride	1 × 2 mg	1 × 2 mg	-
	Isophane insulin	-	-	1 × 8 units
	Neutral insulin	-	-	3 × 6 units
	Allopurinol	1 × 100 mg	1 × 100 mg	1 × 100 mg
	Atorvastatin	1 × 10 mg	1 × 10 mg	1 × 20 mg
Anticoagulative	Acetylsalicylic acid	1 × 75 mg	1 × 75 mg	1 × 75 mg
	Clopidogrel	-	1 × 75 mg	1 × 75 mg

**Table 2:** Cardiac MRI parameters, blood pressure levels and kidney graft function parameters comparison pre-denervation, 1 year after and 5 years after.

	Patient Parameters	Before Denervation	1 Year after	5 Years after
**Cardiac MRI parameters**	Left Ventricular Wall Thickness [mm]	15.8	13.3	12
	Left Ventricular End-Diastolic Volume [mL]	164	151.4	104.9
	Left Ventricular Stroke Volume [mL]	122	121.7	68.4
	End-Diastolic Diameter [cm]	5.4	5.2	4.5
	End-Systolic Diameter [cm]	3.0	2.6	2.6
	Left Ventricular Mass-End Diastolic [g]	230.2	191.7	188.2
	Left Ventricular Mass-End Systolic [g]	256.4	215.6	215.5
	Right Ventricular End-Diastolic Volume [mL]	175	153.2	114.8
	Right Ventricular End-Systolic Volume [mL]	56.3	55.9	49.8
**Ejection fraction (EF)**	EF [%]	74.4	80.4	65.2
**Blood pressure**	Systolic [mmHg]	168	154	163
	Diastolic [mmHg]	88	77	77
**Kidney graft function parameters**	Serum creatinine levels [mg/dL]	1.80	2.05	2.00
	eGFR [mL/kg/min]	41	36	37

Abbreviations: MRI—magnetic resonance imaging, EF—ejection fraction, eGFR—estimated glomerular filtration rate.

**Table 3:** An overview of case reports and trials describing the outcomes of performing renal artery denervation.

Author	Year	Model	Underlying Condition	Main Outcomes
**Bhatt et al.** [8]	2014	Human	Drug-resistant hypertension	Ineffectiveness of the procedure in the overall population; effectiveness proved only in particular groups of patients
**Bhatt et al.** [9]	2022	Human	Drug-resistant hypertension	Better blood pressure management and larger blood pressure reductions in the group of patients after renal denervation compared to patients assigned to sham control—36 months of observation
**Yang et al.** [10]	2013	Human	Heart failure	Reduction in blood pressure values
**Pushpakumar et al.** [11]	2023	Animal	Heart failure	Cardiac hypertrophy reduction, proved cardioprotective character of the procedure
**Huang et al.** [12]	2023	Animal	Heart failure	Cardiac muscle remodeling prevention, antiarrythmic effect
**Yilmaz et al.** [13]	2023	Human	Drug-resistant hypertension	Reduction in blood pressure values
**Kario et al.** [14]	2023	Human	Drug-resistant hypertension	Reduction in blood pressure values, improvement of cardiovascular risk profile
**Sas et al.** [15]	2018	Human	Drug-resistant hypertension	Reduction in blood pressure values, proved safety for a kidney graft
**Stainberg et al.** [16]	2020	Human	Atrial fibrillation with concomitant hypertension	Higher probability of atrial fibrillation recovery
**Bermpeis et al.** [17]	2022	Human	Drug-resistant hypertension	Reduction in blood pressure values

## Data Availability

All data analyzed in the research can be found in the archives of the University Clinical Hospital in Wroclaw.

## Abbreviations

ABPM	ambulatory blood pressure monitoring
CABG	coronary artery bypass grafting
CNi	calcineurin inhibitor
CT	computed tomography
MMF	mycophenolate mofetil
MRI	magnetic resonance imaging
LVH	left ventricular hypertrophy
RFA	radiofrequency ablation
